# Concentrations of Transition Metal Ions in Rat Lungs after Tobacco Smoke Exposure and Treatment with His-Leu Dipeptide

**DOI:** 10.3390/molecules28020628

**Published:** 2023-01-07

**Authors:** Marta Szukalska, Tomasz Frączyk, Ewa Florek, Leszek Pączek

**Affiliations:** 1 Laboratory of Environmental Research, Department of Toxicology, Poznan University of Medical Sciences, 60-631 Poznan, Poland; 2 Institute of Biochemistry and Biophysics, Polish Academy of Sciences, 02-106 Warsaw, Poland; 3 Department of Immunology, Transplantology and Internal Medicine, Medical University of Warsaw, 02-006 Warsaw, Poland

**Keywords:** tobacco smoke, lung, hypertension, metal ions

## Abstract

Tobacco smoking is deleterious to the lungs because it exposes them to many toxic substances. These include transition metal ions, such as cadmium. However, there is a lack of information about the influence of endogenous metal-binding peptides, such as His-Leu (HL), on the lung distribution of transition metals in smokers. To address this, we administered HL subcutaneously to rats exposed to tobacco smoke for six weeks, then we measured the concentrations of transition metal ions in the lungs. We found that exposure to tobacco smoke elevates the concentrations of Cd(II) and Cu(II). Administration of the HL peptide, whose elevation is a consequence of angiotensin receptor blocker anti-hypertension therapy, increases the concentration of Fe in the lungs of rats exposed to smoke. These findings suggest that smoking is a risk factor for patients receiving angiotensin receptor blockers to treat hypertension.

## 1. Introduction

The dipeptide His-Leu (HL) is a co-product of angiotensin I (a peptide with an amino acid sequence: Asp-Arg-Val-Tyr-Ile-His-Pro-Phe-His-Leu) conversion to angiotensin II by angiotensin-converting enzyme (ACE), which cleaves the peptide bond between Phe^8^ and His^9^ residues. Angiotensin II leads to vasoconstriction through binding to the angiotensin II type 1 receptor (AT1R). ACE and AT1R are molecular targets for anti-hypertension therapies [1,2]. Although distributions of angiotensin I and II have been studied thoroughly, that of HL remains unknown. However, one can speculate that the concentration of HL in blood correlates closely with that of angiotensin II because both peptides are products of the same enzymatic cleavage. Inhibition of ACE decreases the concentration of angiotensin II and therapies utilizing ACE inhibitors probably also decrease the concentration of HL. In contrast, angiotensin receptor blockers (ARBs), which antagonize AT1R, trigger a compensatory mechanism that leads to the accumulation of angiotensin II [3,4] and thus probably a concomitant increase in the concentration of HL.

The modulation of HL concentration in the human body is intriguing from the bioinorganic point of view because peptides with an amino-terminal histidine residue can coordinate transition metal ions (e.g., Cu(II) [5,6]) in a bidentate manner via its imidazole and amine nitrogen atoms. Such peptides rapidly form Cu(II) complexes that are labile and redox active [7,8]. Copper ions bound by an N-terminal histidine and another proximal histidine can form a histidine brace motif, which is found in monooxygenases that catalyze polysaccharide oxygenation [9,10,11]. However, HL lacks a second histidine residue and therefore cannot form such a complex unless another peptide or protein acts as a ternary partner. Nevertheless, HL may enable swift transfer of Cu ions between other biomolecules and simultaneously provide a molecular framework for controlled reduction and oxidation of the Cu ion. Such properties may be crucial, as is the case for the acquisition of Cu by the cellular copper transporter CTR1 [12]. Nonetheless, the biological relevance of metal ion complexes formed by HL remains to be discerned.

Dysregulation of transition metal ions, such as Cu(II), leads to many diseases [13,14]. Therefore, the relative abundances of HL and transition metal ions may be important. This is especially true in cases where the concentrations of metal ions in the body are pathological. An example is tobacco smoking, which is known to influence metal concentrations in the body [15,16,17,18,19,20,21,22,23,24]. One of the most frequently reported smoking-related changes is an increase in Cd concentration in lung tissue and blood [15,16,17,18,22]. Changes in metal concentrations in lung tissue may result from their direct transfer from tobacco smoke (most likely the case of Cd and Pb) or their redistribution within the body (more probable for essential metal ions, such as Cu, Fe, and Zn). Such redistribution may be facilitated by metal ion-binding proteins and peptides, such as HL. Thus, it is crucial to know how the concentration of HL, which is potentially elevated during anti-hypertension therapy using ARBs, affects metal ion concentrations after exposure to tobacco smoke. Notably, the amino acid sequences of human and rat angiotensin I and II are identical, making rat models suitable for this type of research. Therefore, we examined the influence of HL on the concentrations of metal ions in the lungs of rats chronically exposed to tobacco smoke.

## 2. Results and Discussion

**Figure 1:**
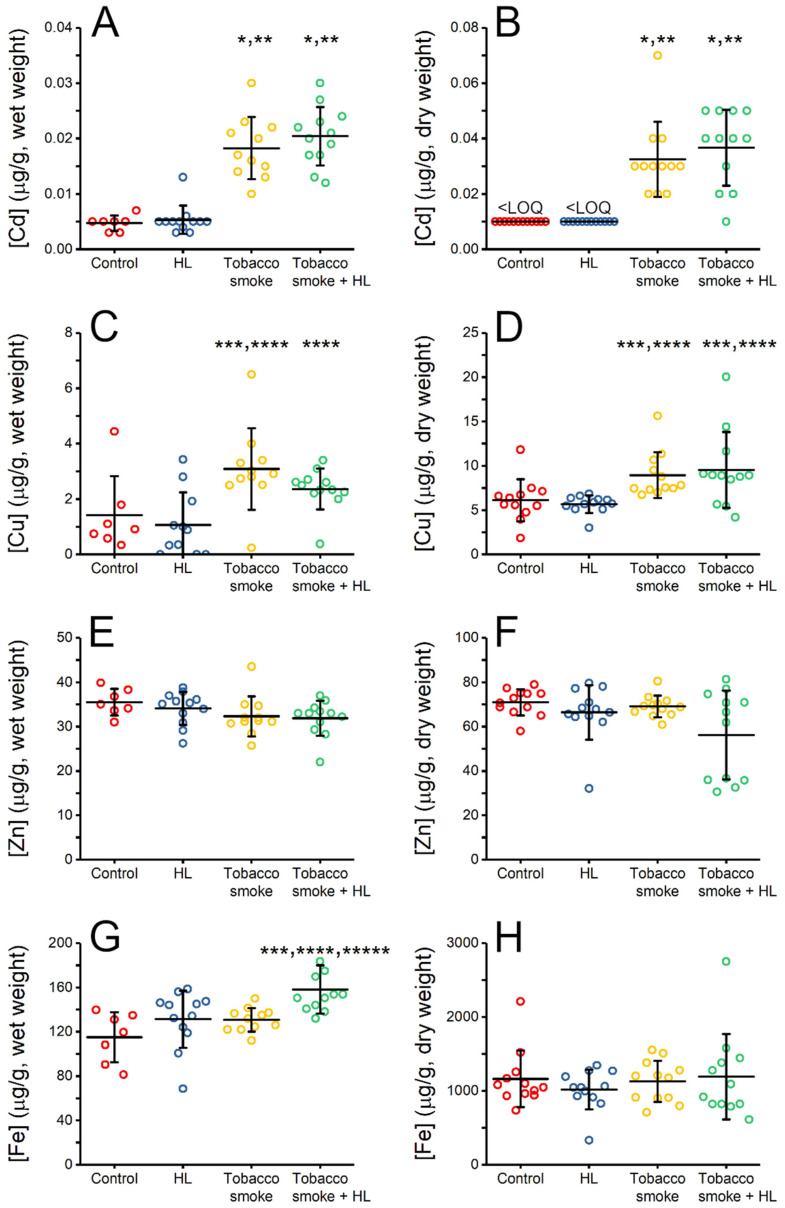
Influence of tobacco smoke exposure and treatment with His-Leu dipeptide (HL) on the concentrations of metals in rat lungs (µg/g), measured in wet (**A**,**C**,**E**,**G**) and dry (**B**,**D**,**F**,**H**) tissue. Shown are all measurement data (circles), mean value (horizontal line), and SD (whiskers). All values for Cd in dry tissue of the “Control” and “HL” groups were below the limit of quantitation (<LOQ; 0.01 µg/g). *p*-values were calculated using a two-sample *t*-test. * *p* < 0.01 vs. “Control” group, ** *p* < 0.01 vs. “HL” group, *** *p* < 0.05 vs. “Control” group, **** *p* < 0.05 vs. “HL” group, ***** *p* < 0.05 vs. “Tobacco smoke” group.

We tested the influence of prolonged exposure to tobacco smoke and treatment with HL on the concentrations of transition metals in the lungs of rats. Measurements were performed in two ways: (i) wet tissue was digested, and metal concentrations were measured directly (Figure 1A,C,E,G); (ii) the tissue was freeze-dried before digestion, and the concentration was expressed per unit dry weight (Figure 1B,D,F,H). This approach improves the power of the conclusions because there are pros and cons of wet and dry tissue processing before metal concentration measurement [25]. Among the variables that may affect the analyses of wet tissue is water content. In contrast, analysis of dry tissue requires an additional freeze-drying step, which may add variability to these sensitive methodologies that can detect metal ions in the ppm-ppb range. 

Metal concentrations in wet lung tissue of control rats, which were not exposed to tobacco smoke, were 0.005 ± 0.001 µg Cd/g, 1.41 ± 1.41 µg Cu/g, 35.5 ± 3.0 µg Zn/g, and 115.0 ± 22.6 µg Fe/g (Figure 1), which compare well with the wet-weight values previously measured in the lugs of healthy Wistar male rats (0.009 ± 0.009 µg Cd/g, 0.71 ± 0.11 µg Cu/g, 11.8 ± 2.1 µg Zn/g, and 35.4 ± 5.0 µg Fe/g [26]). Our results also correspond well with the metal concentrations in the lungs of humans (smokers and non-smokers) [25], where the median dry-weight values were measured to be 0.27 µg Cd/g, 6.02 µg Cu/g, 49.44 µg Zn/g, and 745.56 µg Fe/g [25].

The most pronounced change was observed for Cd (Figure 1A,B), whose concentration in rats exposed to tobacco smoke was four-fold higher than that in non-exposed control rats (*p* < 0.01). Treatment with HL did not change the Cd concentration in the lungs of control rats or those exposed to tobacco smoke. A greater lung concentration of Cd in rats exposed to tobacco smoke compared with that in non-exposed rats was also observed by Dorman et al. [15], and the same result was found for human lungs [16,25]. Cigarettes are the primary source of Cd in the lungs of smokers because of the relatively high levels (µg/g) of this metal in tobacco and its efficient transfer to tobacco smoke [16,24,25,27,28,29,30]. As Cd(II) has similar coordination properties to Zn(II), it can substitute Zn(II) in proteins, such as enzymes and transcription factors. Divalent Cd usually has a higher affinity for proteins than Zn(II), making this substitution favorable. Such substitution commonly has a negative effect on the structure and function of these proteins [31,32,33] and results in lowered protection against oxidative stress [34,35], which leads to DNA damage [36]. Cadmium impairs the innate and adaptive immune systems, by changing cytokine levels or lowering the viability of NK or T cells [37]. Exposure to Cd also increases the risk of lung, prostate, and breast cancers [34,35], and the aforementioned DNA damage and impaired immunity are crucial factors in cancer development.

The second most evident change was observed for Cu, whose concentration in the lungs of rats exposed to tobacco smoke was two-fold higher in wet tissue and 50% higher in dry tissue (*p* < 0.05) than in the lungs of control rats (Figure 1C,D). As observed for Cd, treatment with HL did not change Cu concentration in the lungs of control rats or those exposed to tobacco smoke. Although tobacco contains Cu [38], the higher Cu concentration in the lungs of rats exposed to tobacco smoke may result from the redistribution of this trace metal in the organism, primarily because of smoking-derived inflammation [15,38]. Consistent with our results, the Cu concentration in the blood of smokers was higher than that of non-smokers [39,40]. However, Meral and Akdemir did not find such differences between otherwise-healthy smokers and non-smokers. They suggested that the higher Cu concentration detected in other studies may indirectly result from smoking-related diseases rather than smoking itself [41]. Interestingly, compared with healthy individuals, the serum Cu concentration is also higher in lung cancer patients [42] and esophageal cancer patients [43], although this may be a result rather than the cause of carcinogenesis. Even if a higher concentration of Cu does not cause carcinogenesis, it promotes uncontrolled proliferation of cancer cells because they have a higher demand for Cu than non-dividing cells [44]. Accordingly, Cu depletion appears to impair cancer cell metastasis [45].

No statistically significant differences in the lung concentrations of Zn were observed among the four groups (Figure 1E,F). Analogously, Pinto et al. found no statistically significant differences between the concentrations of Zn in the lungs of smokers and non-smokers [16]. Interestingly, we found that the wet-tissue concentration of Fe in the lungs of HL-treated rats exposed to tobacco smoke (Figure 1G) was significantly higher than that in control rats (*p* < 0.05), rats treated with HL alone (*p* < 0.05), and rats exposed to smoke alone (*p* < 0.05). In freeze-dried tissue, the differences were smaller, and they were not statistically significant at the 0.05 level. Dorman et al. also found that tobacco smoke did not affect the concentration of Fe in rat lungs [15], although the concentration of Fe in the lungs of human smokers was higher than that in non-smokers [23]. Our novel observation that HL-treated rats have a higher lung concentration of Fe when exposed to tobacco smoke highlights the potentially deleterious impact of this endogenous peptide, which is produced during the conversion of angiotensin I to angiotensin II by ACE. This should be taken into account when prescribing anti-hypertension therapies that block angiotensin II binding to AT1R and may thereby lead to increased production of both angiotensin II and HL in the course of compensatory mechanisms. Antlanger et al. have found that ARBs produce a four-fold increase in the blood concentration of angiotensin I and II [3]. Thus, the use of ARBs by smokers may increase the risk of lung diseases resulting from an abnormally high Fe concentration. Notably, Fe homeostasis is disturbed in cancer cells, which show a heightened demand for this transition metal [46,47], and Fe dysregulation is associated with lung cancer initiation and development [48]. Our results suggest that ARBs may increase the risk of cancer in hypertensive patients who smoke. However, more research is needed to examine the correlation between Fe concentrations in the blood and lungs with cancer incidence in smokers treated with ARBs. Recent studies on the influence of ARBs on lung cancer risk are conflicting, with some reporting an increased risk, some revealing no influence, and others reporting a protective action [49,50,51,52,53,54,55]. Some potential pro-carcinogenic actions of ARBs may stem from drug impurities [55,56]. However, they may increase the risk of cancer only in specific circumstances, such as tobacco smoking. Our results suggest the latter.

High concentrations of Cd in blood were found to be associated with high blood pressure [17,18,57,58]. The concentrations of Cd in hypertensive non-smokers are higher than those in normotensive non-smokers and normotensive smokers. Smoking increases the Cd concentrations both in normotensive and hypertensive humans [17]. Cadmium mainly induces oxidative stress and inflammation [58,59,60,61]. Kidney cells accumulate Cd and are damaged by acute exposure [62]. Because the kidney is an essential player in blood pressure regulation, the toxicity of Cd to this organ may lead to hypertension. Furthermore, Cd stimulates ACE activity, leading to a higher concentration of angiotensin II [63]. Thus, smoking is a risk factor for hypertension, which may warrant anti-hypertension therapy. The most common strategy to decrease blood pressure involves treatment with an ACE inhibitor (ACEi) or A1TR blocker (ARB) [64].

**Figure 2:**
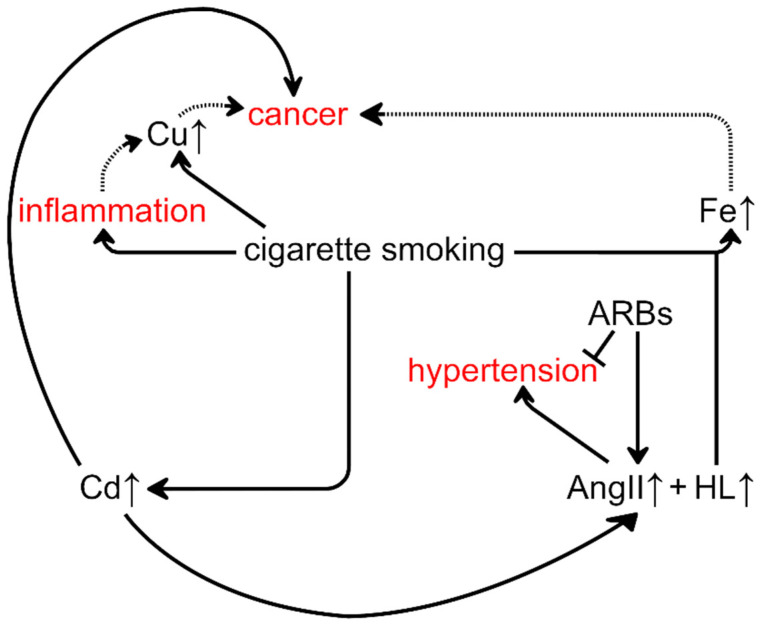
Interdependence of cigarette smoking, hypertension, and cancer, based on this work and that described in the literature. Solid arrows represent confirmed associations and dotted arrows indicate less firmly documented or controversial ones. Cigarette smoking has harmful effects, including carcinogenesis and hypertension. Furthermore, our results show that simultaneous exposure to smoke and HL increases the concentration of Fe in the lungs. The concentration of HL probably increases commensurately with that of angiotensin II (AngII) during anti-hypertension therapy using angiotensin receptor blockers (ARBs). A detailed description of these pathways is provided in the Results and Discussion section.

Our results and the aforementioned interplay between transition metal ions and hypertension and cancer suggest special attention should be given to the potential enhancement of the carcinogenic action of smoking by ARB-based therapy targeting hypertension (Figure 2). Through Cd exposure, smoking may cause cancer and hypertension [17,35,58,65,66]. Treatment of hypertension by ARBs may lead to an increased concentration of HL, which increases the concentration of Fe in the lungs of rats exposed to tobacco smoke. Disruption of Fe metabolism increases the risk of carcinogenesis [67]. Thus, ARBs may potentiate the carcinogenesis initiated by tobacco smoke. This hypothesis is consistent with the research of Opelz and Döhler, who showed that ACEi and ARBs treatment only increases the risk of malignant tumors in patients currently smoking or with a history of smoking [68]. However, our results indicate that it may be safer to treat hypertension in smokers using an ACEi that may decrease the HL concentration rather than ARB that may increase it. This issue should be carefully explored because both drug classes have been recently proposed not only as anti-hypertensives but also as potential adjuvants for anti-cancer therapy [69,70,71,72]. Furthermore, they may be beneficial as an add-on treatment for COVID-19 [73] and may lower the risk of developing neurodegenerative conditions, such as Parkinson’s and Alzheimer’s disease [74,75].

## 3. Materials and Methods

### 3.1. Ethics Committee Approval

The study design was approved by the Ethics Committee for Animal Experiments Affairs in Poznan, Poland. All procedures concerning the handling and use of laboratory animals were performed in accordance with European Union regulations under Directive 2010/63/EU on the protection of animals used for scientific purposes and followed the principle of replacement, reduction, and refinement. The number of animals and observation time used in this study satisfied the minimum required to reliably detect the expected effect size. To improve the rigor and reproducibility of animal research, all data were collected according to ARRIVE 2.0 guidelines. In vivo experiments were carried out in the animal house of the Department of Toxicology at the Poznan University of Medical Sciences (Poznan, Poland). Contractors had individual permits for planning and performing experiments and sacrificing animals.

### 3.2. Animals

Wistar male rats were bred in the Department of Toxicology at the Poznan University of Medical Sciences (Poznan, Poland). The research was carried out on 48 male rats (3 months old) with a mean body weight of 360 ± 20 g. The animals were kept in polypropylene cages (two rats per cage) with a floor area of 1820 cm^2^ and height of 20 cm, which were housed in a room with standardized lighting (12 h light/12 h dark cycle), humidity (50–60%), and temperature (22 ± 2 °C). Animals had continuous access to food (Labofeed B standard) and sterilized drinking water. Labofeed B was purchased from the “Morawski” Feed Production Plant (Kcynia, Poland) and has a dietary formula based on the recommendations of the National Research Council (US) Subcommittee on Laboratory Animal Nutrition [76]. Animals were allowed to acclimatize for two weeks before the experiment, then observed daily and weighed once per week throughout the experiment.

### 3.3. Experimental Design

**Figure 3:**
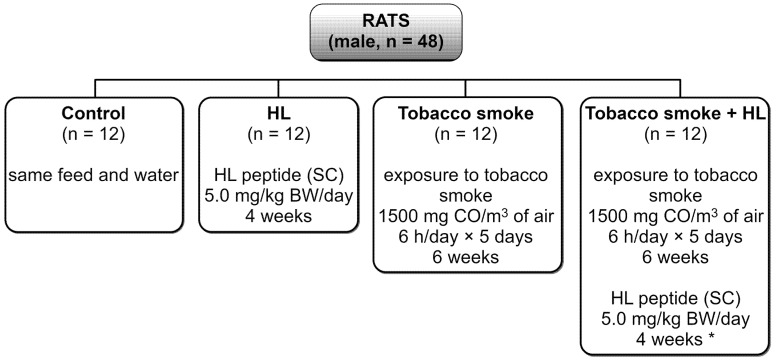
Experimental design of the in vivo study. HL was administered by subcutaneous (SC) injection. * HL was administered starting from the third week of exposure to tobacco smoke.

Sexually mature male rats were randomly divided into four experimental groups (twelve animals per group), as shown in Figure 3. Exposure to tobacco smoke was carried out for six weeks. Treatment with HL (administered by subcutaneous injection, analogously to angiotensin II administration in other studies [77,78,79,80,81,82,83,84]) started from the third week of exposure to tobacco smoke and lasted four weeks.

HL was obtained from Sigma-Aldrich. The following rationale was a basis for the choice of dosage of HL. We assumed that the concentration of HL correlates closely with that of angiotensin II because both peptides are products of the same enzymatic cleavage. The concentration of angiotensin II in human blood is approximately 10 pM [85,86]. The half-life of this peptide in pig blood is approximately 0.5 min, and in the heart, kidney, and adrenal tissues, it is approximately 15 min [87]. Administering 5 mg HL/kg body weight per day would result in the basal concentration (10 pM) in the whole rat body after about four hours following injection, assuming a 10 min half-life of HL because of the subcutaneous route (in contrast to intravenous injection) and the average rat tissue water content of 65.6% [88]. Although the chosen dosage may seem relatively low, angiotensin II is administered to rats usually subcutaneously in the concentration range of 0.1–1.5 mg/kg body weight per day, with biological effects observed on the level of the whole organism [77,78,79,80,81,82,83,84]. Therefore, our choice for dosage is higher than that of angiotensin II (calculating it, one should also consider the difference in molecular weights of these peptides, which means that we administered from 14- to 190-fold higher molar content of HL than angiotensin II administered in other studies). However, we should note that we administered HL once per day, opposite to angiotensin II, which was usually administered constantly using an osmotic mini-pump [77,78,79,80,81,82,83,84].

Animals were exposed to tobacco smoke from unfiltered cigarettes (Imperial Tobacco Polska S.A., Tarnowo Podgorne, Poland) containing 10 mg tar, 0.9 mg nicotine, and 8 mg carbon monoxide (CO). The tobacco products were placed in a combustion scrubber and the content of CO in the air of the chamber was controlled using a CO sensor with a patented auto-calibration procedure. The oxygen level was maintained at 20 ± 0.5% of the air volume. Air in the chamber was changed ten times per day [89]. 

Animals in the “Tobacco smoke” group and “Tobacco smoke + HL” group were exposed to tobacco smoke containing 1500 mg CO/m^3^, six hours per day, five days per week, for six weeks. Right after the last exposure, rats were anesthetized using intramuscular administration of ketamine (40 mg/kg body weight) and xylazine (5 mg/kg body weight) and then autopsied. The left lung (upper lobe) was stored in sterile tubes at −80 °C until elemental analysis was performed.

### 3.4. Mineralization and Elemental Analysis

Samples of wet lung tissue (0.2–0.5 g) were digested in a microwave mineralizer containing 8 mL 65% *w/w* HNO_3_. Using an Agilent 200 Series atomic absorption spectrometer (Agilent Technologies, Santa Clara, CA, USA), atomic absorption spectrometry was used to measure the Cd concentration and flame atomic absorption spectrometry was used to measure the concentrations of Cu, Zn, and Fe. Acquisition parameters for Cd measurement were: wavelength, 228.8 nm; slit width, 0.5 nm; lamp current, 4.0 mA; atomization temperature, 1800 °C in argon. The acquisition parameters for Cu, Zn, and Fe measurements were: wavelength, 324.8 nm (Cu), 213.9 nm (Zn), or 248.3 nm (Fe); slit width, 0.5 nm (Cu), 1.0 nm (Zn), or 0.2 nm (Fe); lamp current, 4.0 mA (Cu) or 5.0 mA (Zn, Fe); flame, mixture of air (13.5 L/min) and acetylene (2.0 L/min). Analysis of Cd, Cu, Zn, and Fe concentrations in dry tissue (0.1–0.2 g) was performed using an Agilent 7500a inductively coupled plasma mass spectrometer (Agilent Technologies, Santa Clara, CA, USA) after freeze-drying and digestion of samples in a 25 mL mixture of 13% *w/w* HNO_3_ and 0.6% *w/w* H_2_O_2_. A plasma power of 1310 W was used, and the internal standard was ^89^Y.

## 4. Conclusions

The lung concentrations of Cd and Cu in rats exposed to tobacco smoke are higher than those in non-exposed rats. Administration of HL, a co-product of angiotensin I to angiotensin II conversion by ACE, does not change the Cd and Cu concentrations. However, HL administration does increase the concentration of Fe in the lungs of rats exposed to tobacco smoke. ARBs used in anti-hypertension therapies probably increase the concentration of endogenous HL. Consequently, ARBs may raise the risk of metal-induced carcinogenesis in hypertensive smokers. Therefore, more information is needed about the HL distribution in the human body, especially during treatment with ARBs. In addition, the impact of potential changes in HL concentration in the human body on Fe concentration is also of great interest. Such knowledge may help to improve anti-hypertensive therapies for smoking patients.

Several other points need further research. Among them is the molecular mechanism of the influence of HL on the concentrations of transition metals. A clarification is needed on whether the effects observed in our study occur because of the direct binding of metal ions by HL in the lungs or through redistribution of them from other parts of the body. Additionally, an indirect influence of HL on transition metal trafficking cannot be excluded. Furthermore, spatial mapping of transition metal distribution within lung tissue exposed to tobacco smoke would reveal if the results presented here are relevant for intracellular or extracellular parts and to what extent they are the consequence of the physical attachment of tobacco smoke components to the respiratory epithelium.

## Data Availability

The data presented in this study are available on request from the corresponding author.

## Sample Availability

Samples of the compounds used in this study are available on request from the authors.
